# Supplementary material for: Both Nodal signalling and stochasticity select for prospective distal visceral endoderm in mouse embryos
Source: Nat Commun. 2017 Nov 14;8:1492. doi: 10.1038/s41467-017-01625-x (PMC5686177; doi:10.1038/s41467-017-01625-x)
Supplement: Supplementary file 1 — Supplementary Information [file 41467_2017_1625_MOESM1_ESM.pdf]

SUPPLEMENTARY INFORMATION

Supplementary Table 1. Primary and secondary antibodies used in this study.

Antibodies	Species	Company	Catalog number	Dilution
Primary				
Oct3/4	Rabbit	MBL	PM048	1/200
Sox2	Goat	R&D Systems	AF2018	1/100
GATA6	Goat	R&D Systems	AF1700	1/100
RFP	Rabbit	MBL	PM005	1/100
EGFP	Rabbit	MBL	#598	1/100
EGFP	Rat	Nacalai	#04404-84	1/100
Lefty	Goat	R&D Systems	AF746	1/50
Cerl1	Rat	R&D Systems	225807	1/50
Nodal	Rabbit	Abcam	Ab39953	1/50
Secondary				
Alexa 488 anti–rabbit IgG	Donkey	Molecular Probes	A21206	1/200
Alexa 568 anti–rat IgG	Donkey	Molecular Probes	A11057	1/200
Alexa 594 anti–rat IgG	Donkey	Molecular Probes	A21209	1/200
Alexa 633 anti–goat IgG	Donkey	Molecular Probes	A21082	1/200

Supplementary Table 2. The numbers of PrE cells and DVE cells in *Lefty* mutant embryos.

(a)

Genotype	DAPI (cells)	PrE (cells)	*Lefty1(mVenus)* (cells)
Lefty1$^{+/+}$	125	40	4
	136	40	4
	134	41	5
Lefty1 $^{+/-}$	131	36	4
	145	49	7
	132	48	7
	99	29	5
	132	43	7
	128	37	10
Lefty1$^{-/-}$	117	34	9
	130	37	6
	138	48	16
	100	35	3
	113	51	10
	116	24	4

(b)

Genotype	DAPI (cells)	PrE (cells)	*Lefty1(mVenus)* (cells)
Lefty1,2 $^{+/+}$	97	26	3
	146	45	5
	128	34	5
	94	23	3
	133	29	4
	132	29	6
	112	34	3
Lefty1,2 $^{+/-}$	145	41	9
	149	48	10
	138	40	6
	146	46	9
	88	24	6
	123	33	6
	92	19	4
	137	36	5
	101	29	7
	104	28	5
	131	34	5
	143	38	4
	144	32	7
	131	44	7
	117	31	7
	143	44	9
	143	46	5
	137	43	5
	153	47	7
	135	37	7
	137	36	8
	184	54	12
	176	44	12
	170	46	13
	146	31	6
	110	32	4
Lefty1,2 $^{-/-}$	147	43	16
	122	38	14
	148	33	16
	147	46	13
	101	30	7
	138	40	15

(c)

Genotype	DAPI+ (cells)	oct3/4+ (cells)
Lefty1,2 $^{+/+}$	149	58
	122	39
Lefty1,2 $^{+/-}$	125	30
	124	48
	136	42
	151	32
	123	43
	148	30
	124	45
Lefty1,2 $^{-/-}$	135	43
	132	40
	117	31
	130	44

Footnotes to Table 2: The numbers of DAPI^{+} cells, PrE cells, mVenus^{+} and Oct3/4^{+} cells are summarized for individual embryos with the indicated genotype. The Table corresponds to Figure 4.

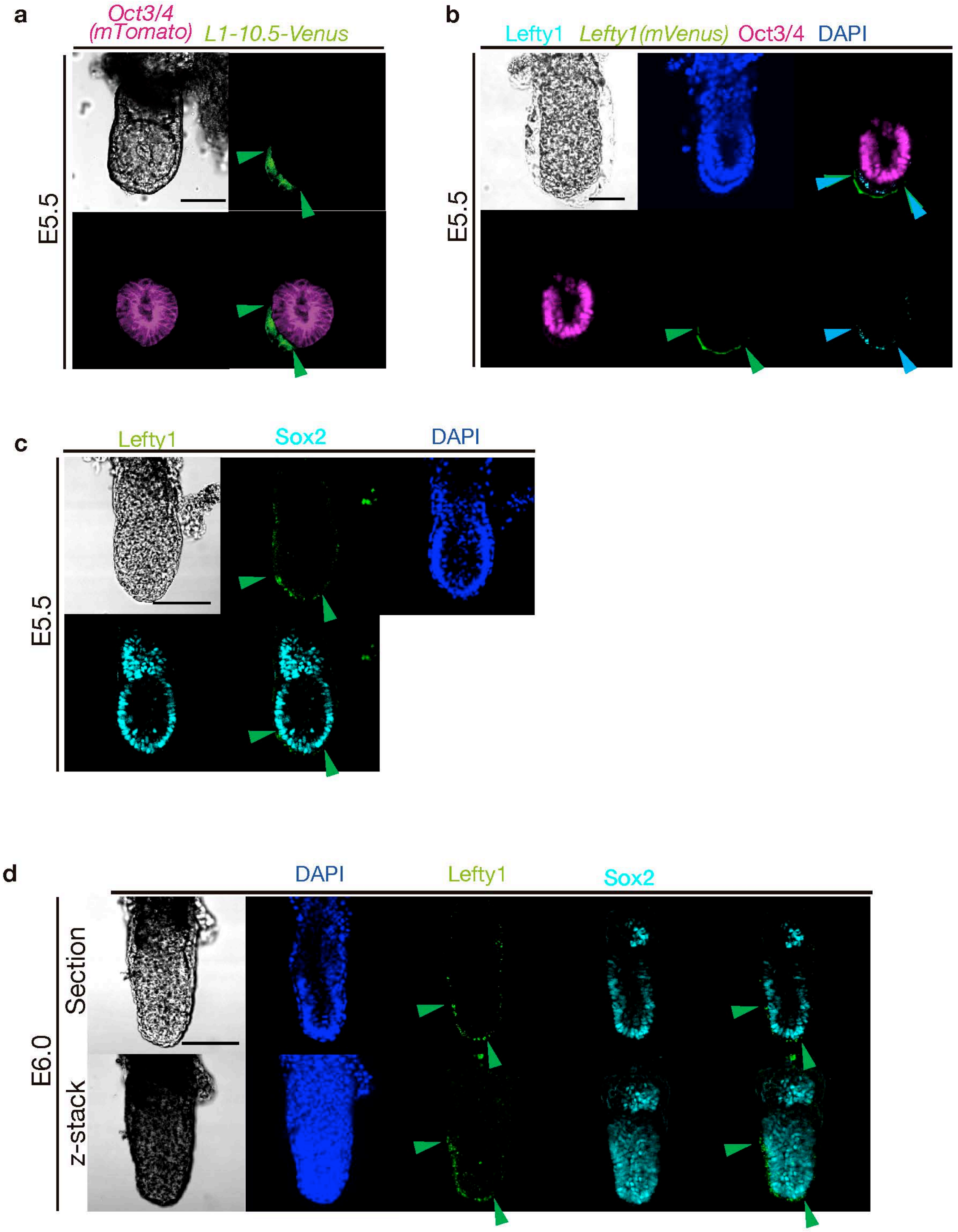
a
Oct3/4
(mTomato)
L1-10.5-Venus
E5.5
b
Lefty1
Lefty1(mVenus)
Oct3/4
DAPI
E5.5
c
Lefty1
Sox2
DAPI
E5.5
d
DAPI
Lefty1
Sox2
E6.0
Section
z-stack

Supplementary Figure 1. Epiblast does not contribute to DVE. (**a**) An E5.5 mouse embryo harboring *L1-10.5-Venus* and *Oct3/4(mTomato)* BAC transgenes were examined for Venus and mTomato immuno-fluorescence. Note that epiblast cells (marked with mTomato expressed under the control of the *Oct3/4* promoter) do not contribute to DVE (labeled with Venus expressed under the control of the 10.5-kb upstream region of *Lefty1*). Arrowheads indicate DVE region. (**b**) An E5.5 embryo harboring *Lefty1(mVenus)* was examined for Venus, endogenous Lefty1 and Oct3/4 immuno-fluorescence. Note that Lefty1^{+} cells and Oct3/4^{+} cells are segregated. (**c**) An E5.5 wild-type embryo was stained for endogenous Lefty1 and Sox2. (**d**) An E6.0 wild-type embryo was immunostained for endogenous Lefty1 and Sox2. Sox2^{+} cells are negative for Lefty1. All scale bars, 200 µm.

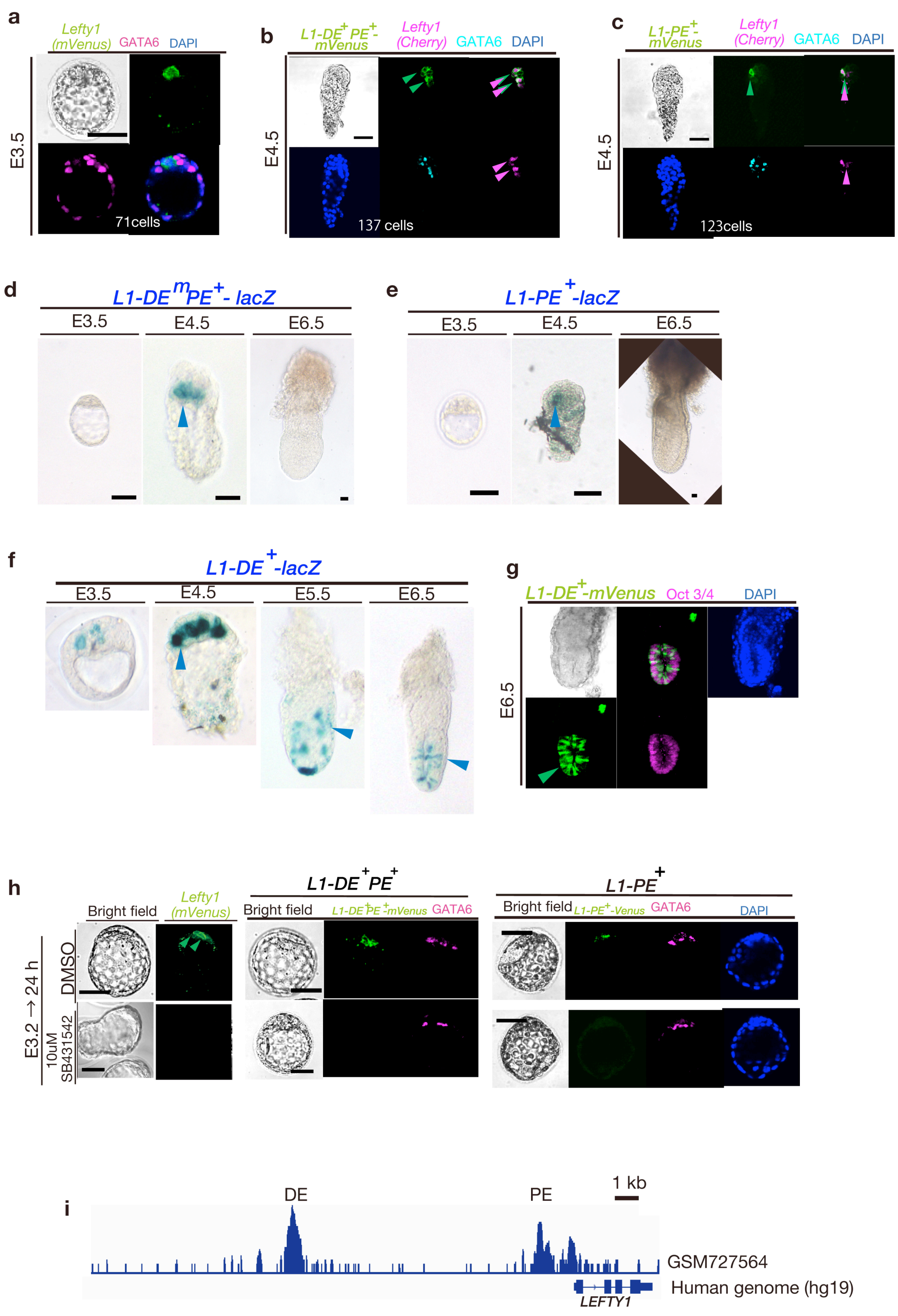
a
Lefty1 (mVenus)
GATA6
DAPI
E3.5
71cells
b
L1-DE+ PE+-mVenus
Lefty1 (Cherry)
GATA6
DAPI
E4.5
137 cells
c
L1-PE+-mVenus
Lefty1 (Cherry)
GATA6
DAPI
E4.5
123cells
d
L1-DEmPE+- lacZ
E3.5
E4.5
E6.5
e
L1-PE+-lacZ
E3.5
E4.5
E6.5
f
L1-DE+-lacZ
E3.5
E4.5
E5.5
E6.5
g
L1-DE+-mVenus
Oct 3/4
DAPI
E6.5
h
Bright field
Lefty1 (mVenus)
L1-DE+PE+
Bright field
L1-DE+PE+-mVenus
GATA6
L1-PE+
Bright field
L1-PE+-Venus
GATA6
DAPI
E3.2 → 24 h
DMSO
10uM SB431542
i
DE
PE
1 kb
GSM727564
LEFTY1
Human genome (hg19)

Supplementary Figure 2. ***Lefty1*** **expression in L1^{epi} cells and L1^{dve} cells is regulated by Nodal-Foxh1 signaling.** (**a**) An E3.5 embryo harboring a *Lefty1(mVenus)* BAC transgene was examined for mVenus and GATA6 expression by immunofluorescence analysis. Scale bar, 50 μm. Note that the mVenus^{+} cell (L1^{epi} cell) is negative for GATA6. (**b**) An E4.5 embryo harboring *L1-DE^{+}PE^{+}-mVenus* and *Lefty1(Cherry)* BAC transgenes was examined for mVenus, Cherry, and GATA6 immunofluorescence. Note that Venus^{+} cells at this stage (L1^{dve} cells) are positive for Cherry and GATA6 (arrowheads). Scale bar, 50 μm. (**c**) An E4.5 embryo harboring *L1-PE^{+}-mVenus* and *Lefty1(Cherry)* BAC transgenes was examined for mVenus, Cherry, and GATA6 immunofluorescence. Note that mVenus^{+} cells at this stage (L1^{dve} cells) are positive for Cherry and GATA6. Scale bar, 50 μm. (**d–f**) Embryos harboring *L1-DE^{m}PE^{+}-lacZ* (**d**), *L1-PE^{+}-lacZ* (**e**), or *L1-DE^{+}-lacZ* (**f**) were stained with X-gal at the indicated stages. Arrowheads indicate X-gal + cells in EPI. (**g**) An E6.5 embryo harboring *L1-DE^{+}Venus* was examined for Venus and endogenous *Oct3/4(mTomato)*. Note that Venus^{+} cells are positive in EPI cells (Oct3/4^{+}). Arrowheads indicate Venus + cells in EPI. (**h**) E3.2 embryos harboring *Lefty1(mVenus)*, *L1-DE^{+}PE^{+}-mVenus*, or *L1-PE^{+}-Venus* transgenes were cultured for 24 h with 10 μM SB431542 (Sigma #S4317) or dimethyl sulfoxide (DMSO) vehicle and were then examined for GATA6 as well as mVenus or Venus immune-fluorescence. Arrowheads indicate transgene expression in L1^{epi} cells of control embryos. Scale bars, 50 μm. (**i**) Localization of Foxh1 binding sites in the *LEFTY1* gene of human embryonic stem cells by ChIP-seq analysis with antibodies to Foxh1[#1]. The Foxh1 binding peaks are located at the DE and PE regions.

a

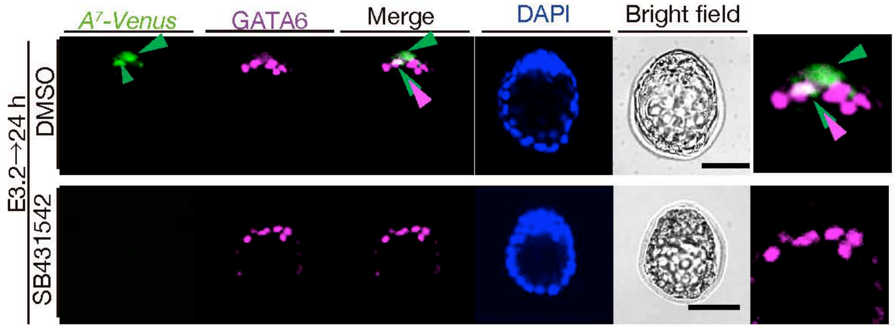

b

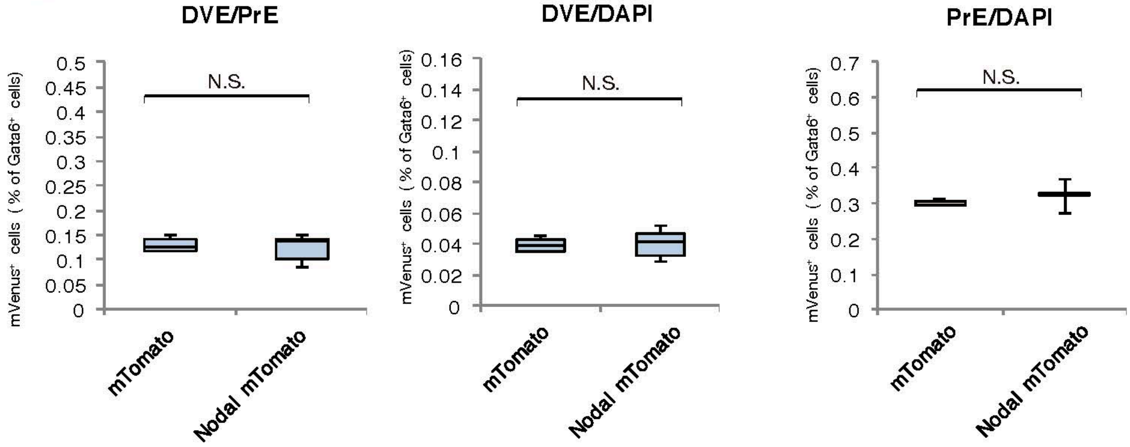

c

injected mRNA	DAPI(cells)	PrE (cells)	*Lefty1(mVenus)*(cells)
mTomato	142	42	5
	118	35	5
	131	40	6
	143	42	5
	128	40	5
Nodal mRNA + mTomato mRNA	135	50	7
	140	46	4
	154	50	5
	129	42	6
	121	33	5

Supplementary Figure 3. Nodal-Foxh1 signaling activity as revealed with the *A_7-Venus* transgene. (**a**) E3.2 embryos harboring the *A_7-Venus* transgene were cultured for 24 h in the presence of 10 μM SB431542 or DMSO vehicle and were then subjected to immunofluorescence staining for Venus and GATA6 as well as to staining of nuclei with DAPI. Venus$^+$ cells in the control embryo are either GATA6$^+$ or GATA6$^-$ (arrowheads), presumably corresponding to L1^{dve} and L1^{epi} cells, respectively. Note that Venus expression is abolished by SB431542. Scale bars, 50 µm. (**b**) Data are presented as box-and-whisker plots(first and third quartile, the line represents the median; whiskers: minimum to maximum). The number of DVE cells as a percentage of PrE cells, the number of DVE cells as a percentage of total cells and the number of PrE cells as a percentage total cells cells are summarized for embryos harboring a *Lefty1(mVenus)* BAC transgene, injected with *mTomato* mRNA with or without *Nodal* mRNA. NS, not significant (*t*-test).. (c) the numbers of DAPI$^+$ cells, PrE cells, and mVenus$^+$ cells are summarized for *Nodal* mRNA-injected and *mTomato* mRNA-injected embryos.

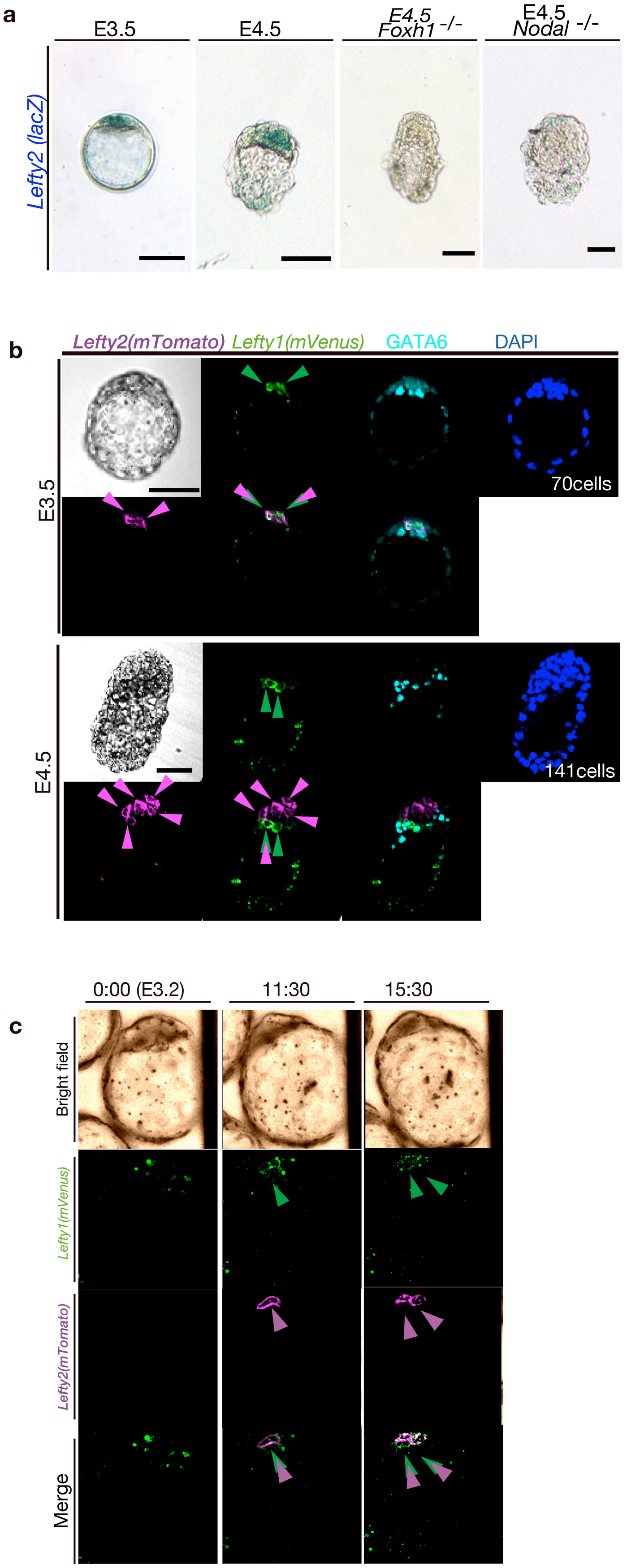

a
E3.5
E4.5
E4.5 Foxh1 -/-
E4.5 Nodal -/-
Lefty2 (lacZ)
b
Lefty2(mTomato)
Lefty1(mVenus)
GATA6
DAPI
E3.5
70cells
E4.5
141cells
c
0:00 (E3.2)
11:30
15:30
Bright field
Lefty1(mVenus)
Lefty2(mTomato)
Merge

Supplementary Figure 4. ***Lefty2*** **expression overlaps in part with that of** ***Lefty1*** **and is regulated by Nodal-Foxh1 signaling.** (**a**) Expression of *Lefty2* as monitored with a *Lefty2(lacZ)* BAC transgene. *Lefty2* was expressed in the ICM at E3.5 and in the epiblast at E4.5. Such expression of the transgene was lost in *Foxh1*$^{-/-}$ or *Nodal*$^{-/-}$ embryos. Scale bars, 50 µm. (**b**) Embryos harboring both *Lefty1(mVenus)* and *Lefty2(mTomato)* BAC transgenes were examined for mVenus, mTomato, and GATA6 expression at E3.5 and E4.5. Note that *Lefty1* and *Lefty2* are expressed in the same cells at E3.5 (arrowheads indicated mTomato + cells in magenta and mVenus+ cells in green). At E4.5, *Lefty1* is expressed in GATA6^{+} cells whereas *Lefty2* is expressed mostly in GATA6^{-} cells. Scale bars, 50 μm. (**c**) An E3.2 embryo harboring both *Lefty1(mVenus)* and *Lefty2(mTomato)* BAC transgenes was cultured for 15.5 h. Fluorescence of mVenus and mTomato was recorded at the indicated times. Note that *Lefty1* and *Lefty2* are coexpressed at the indicated times in a subset of ICM cells (arrowheads).

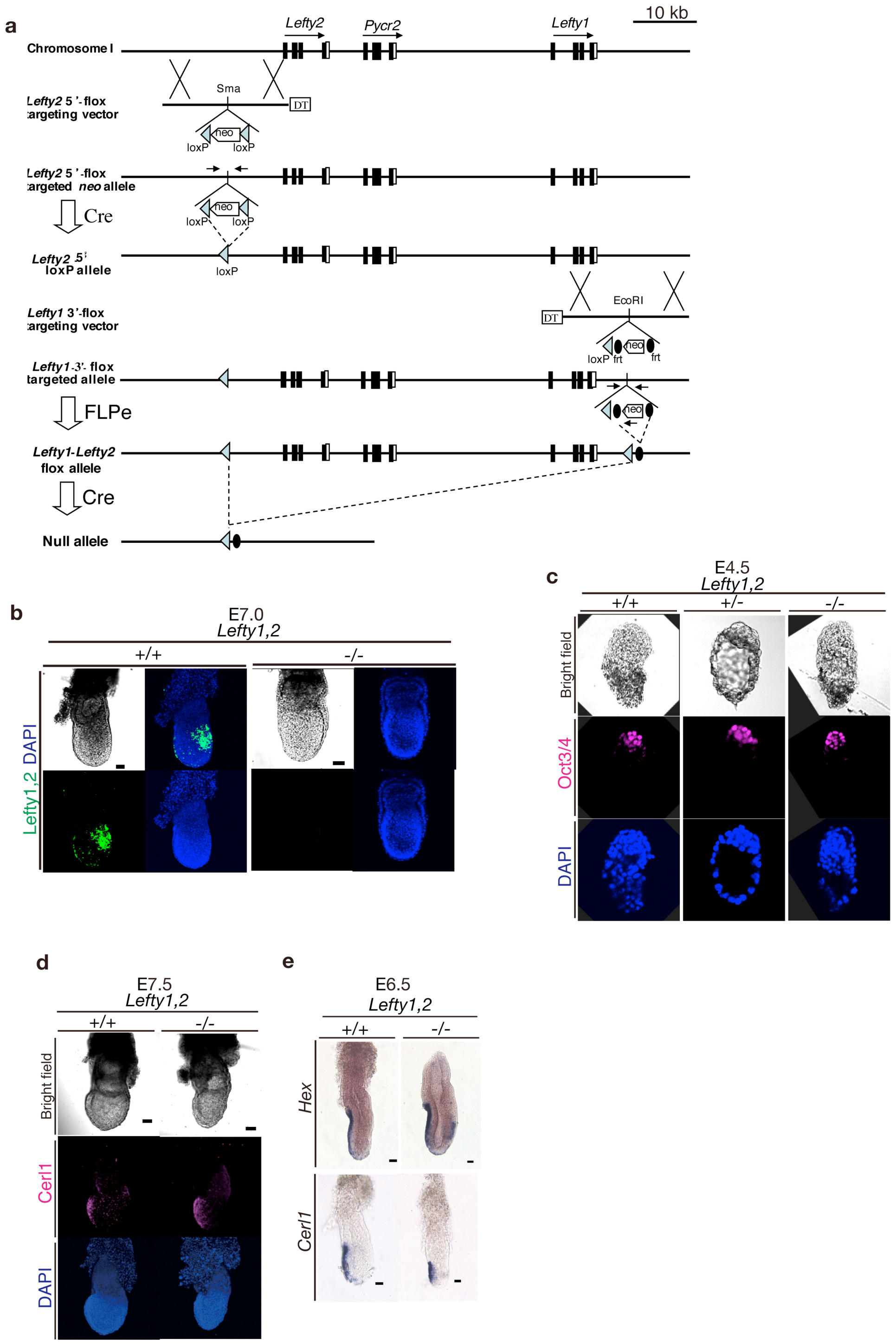
a
10 kb
Chromosome I
Lefty2
Pycr2
Lefty1
Lefty2 5'-flox targeting vector
Sma
DT
neo
loxP
Lefty2 5'-flox targeted neo allele
Cre
Lefty2 5' loxP allele
Lefty1 3'-flox targeting vector
EcoRI
frt
Lefty1 3'-flox targeted allele
FLPe
Lefty1-Lefty2 flox allele
Null allele
b
E7.0
Lefty1,2
+/+
-/-
Lefty1,2 DAPI
c
E4.5
+/-
Bright field
Oct3/4
DAPI
d
E7.5
Cerl1
e
E6.5
Hex

Supplementary Figure 5. Generation of *Lefty1,2*$^{-/-}$ mice and development of the A-P axis in such embryos at E6.5 and E7.5. (**a**) Strategy for generation of a *Lefty1,2* double-mutant allele. (**b**) *Lefty1,2*$^{+/+}$ and *Lefty1,2*$^{-/-}$ embryos at E7.0 were stained with antibodies that recognize both Lefty1 and Lefty2 proteins. Note that Lefty proteins are absent in the *Lefty1,2*$^{-/-}$ embryo. Scale bars, 50 µm. (**c**) *Lefty1,2*$^{+/+}$, *Lefty1,2*$^{+/-}$, and *Lefty1,2*$^{-/-}$ embryos at E4.5 were stained for Oct3/4. Note that Oct3/4^{+} cells are maintained in the *Lefty1,2*$^{-/-}$ embryo. (**d**) *Lefty1,2*$^{+/+}$ and *Lefty1,2*$^{-/-}$ embryos at E7.5 were subjected to immunofluorescence staining for Cerl1 (a marker for AVE). Scale bars, 50 µm. (**e**) Expression of *Cerl1* and *Hex* in E6.5 *Lefty1,2*$^{+/+}$ and *Lefty1,2*$^{-/-}$ embryos as determined by *in situ* hybridization. Scale bars, 50 µm.

Supplementary References

#1. Kim, S.W. *et al*. Chromatin and transcriptional signatures for Nodal signaling during endoderm formation in hESCs. *Dev Biol* **357**, 492-504 (2011).
