# Supplementary material for: Both Nodal signalling and stochasticity select for prospective distal visceral endoderm in mouse embryos
Source: Nat Commun. 2017 Nov 14;8:1492. doi: 10.1038/s41467-017-01625-x (PMC5686177; doi:10.1038/s41467-017-01625-x)
Supplement: Supplementary file 3 — Description of Additional Supplementary Files [file 41467_2017_1625_MOESM3_ESM.docx]

**Description of Additional Supplementary Files**

File Name: Supplementary Movie 1

Description: Time-lapse movie showing the relation between Nodal-Foxh1 signalling and Lefty1 expression. The z-stack movie corresponds to Figure 2c. An E3.2 embryo harboring A7-Venus and a Lefty1(Cherry) BAC transgene was cultured and monitored for Venus (green) and Cherry (magenta) fluorescence by time-lapse microscopy for 9 h.

File Name: Supplementary Movie 2

Description: Time-lapse movie showing the relation between Nodal-Foxh1 signalling and Lefty1 expression in the presence of SB431542. The z-stack movie corresponds to Figure 2d. An E3.2 embryo harboring A7-Venus and a Lefty1(Cherry) BAC transgene was cultured with 10 µM SB431542 and monitored for Venus (green) and Cherry (magenta) fluorescence by time-lapse microscopy for 23 h and 15 min.

File Name: Supplementary Movie 3

Description: Time-lapse movie showing the relation between Nodal and Lefty1 expression. The z-stack movie corresponds to Figure 3a. An E3.2 embryo harboring Lefty1(mVenus) and Nodal(Tomato) BAC transgenes was cultured and monitored for mVenus (green) and Tomato (magenta) fluorescence by time-lapse microscopy for 4 h and 45 min.

File Name: Supplementary Movie 4

Description: Time-lapse movie showing the relation between Lefty1 and Lefty2 expression. The z-stack movie corresponds to Supplementary Figure 4c. An E3.2 embryo harboring Lefty1(mVenus) and Lefty2(mTomato) BAC transgenes was cultured and monitored for mVenus (green) and mTomato (magenta) fluorescence by time-lapse microscopy for 16 h and 15 min.
